# Beyond rotamers: a generative, probabilistic model of side chains in proteins

**DOI:** 10.1186/1471-2105-11-306

**Published:** 2010-06-05

**Authors:** Tim Harder, Wouter Boomsma, Martin Paluszewski, Jes Frellsen, Kristoffer E Johansson, Thomas Hamelryck

**Affiliations:** 1 The Bioinformatics Section, Department of Biology, University of Copenhagen, Copenhagen, Denmark; 2 DTU Elektro, Technical University of Denmark, Lyngby, Denmark; 3 Department of Chemistry, University of Cambridge, Cambridge, UK; 4 Section for Biomolecular Sciences, Department of Biology, University of Copenhagen, Copenhagen, Denmark

## Abstract

**Background:**

Accurately covering the conformational space of amino acid side chains is essential for important applications such as protein design, docking and high resolution structure prediction. Today, the most common way to capture this conformational space is through rotamer libraries - discrete collections of side chain conformations derived from experimentally determined protein structures. The discretization can be exploited to efficiently search the conformational space. However, discretizing this naturally continuous space comes at the cost of losing detailed information that is crucial for certain applications. For example, rigorously combining rotamers with physical force fields is associated with numerous problems.

**Results:**

In this work we present BASILISK: a generative, probabilistic model of the conformational space of side chains that makes it possible to sample in continuous space. In addition, sampling can be conditional upon the protein's detailed backbone conformation, again in continuous space - without involving discretization.

**Conclusions:**

A careful analysis of the model and a comparison with various rotamer libraries indicates that the model forms an excellent, fully continuous model of side chain conformational space. We also illustrate how the model can be used for rigorous, unbiased sampling with a physical force field, and how it improves side chain prediction when used as a pseudo-energy term. In conclusion, BASILISK is an important step forward on the way to a rigorous probabilistic description of protein structure in continuous space and in atomic detail.

## Background

With the emergence of the first experimentally determined protein structures, the investigation of the conformations of their amino acid side chains began. It quickly became apparent that the *χ *(chi) dihedral angles - the main degrees of freedom in side chains - are not distributed freely, but tend to cluster around certain positions [1-3]. This behavior was already well known for dihedral bond angles in small organic molecules [4]. As more protein structures were solved to a high resolution, an increasingly accurate analysis of the adopted conformations became possible.

Today, the most common way to describe the conformational space of side chains is through so-called rotamer libraries: collections of representative side chain conformations, called rotamers [5]. These libraries are usually compiled from experimentally determined, high resolution protein structures by clustering the side chain conformations. Clusters of side chain angles thus obtained can then for example be represented by Gaussian distributions [6,7]. Traditionally, a rotamer in a library corresponds to the mean conformation of such a cluster and may be interpreted as a local energy minimum [5]. In recent studies, rotamer libraries with many thousands of rotamers were used [8,9]. Such libraries are built to cover the conformational space with high resolution.

The accurate description of side chain conformational space is an important ingredient of the solution to many biomolecular problems, such as protein design, docking and high resolution protein structure prediction. Over the past decades rotamer libraries have been successfully applied in all of these contexts. Arguably, the most well studied field is the prediction of side chain conformations for a fixed protein backbone; there is a manifold of programs in the public domain devoted to this problem [10-14].

Rotamer libraries are usually applied as discrete collections of side chain conformations. Discretizing conformational space is a popular approximation to solve problems that are computationally expensive otherwise. For example, when assigning side chains in the densely packed protein core, discretizing the search space leads to exhaustive yet fast search strategies (such as dead end elimination) which can quickly find a global minimum solution [10,15,16]. However, in recent studies it was shown that a Markov chain Monte Carlo (MCMC) sampling scheme combined with a detailed rotamer library yields equally good or better results, which suggests that the combinatorial problem often poses no significant obstacle to finding a global minimum solution [8,13].

The discretization in rotamer libraries inherently leads to edge effects [17,18]. In docking problems for example, small differences in the side chain conformation may result in large differences in energy. Rotamers are often used as stiff building blocks; not considering the conformations in between rotamers may skip energetically favorable configurations [17,18]. There are three common heuristic strategies to tackle these issues. The first, which is most commonly used, is tweaking the energy functions; for example, by reducing the influence of mild steric clashes [11,13,19]. The second is to increase the number of rotamers in the library in order to improve its resolution [8,9,19]. Finally, one can combine rotamer based sampling with some form of continuous optimization [3,17]. Another problem associated with rotamer libraries is the occurrence of so-called non-rotameric states: side chain conformations that cannot be assigned to any of the standard gauche+, gauche- or trans states [7]. Such conformations are typically missing in rotamer libraries and difficult to fill in correctly [20,21].

Here we present BASILISK, a dynamic Bayesian network [22] that formulates a probabilistic model of the conformational space of amino acid side chains. The model is generative: it allows sampling of plausible side chain conformations. BASILISK loosely stands for ***Ba****yesian network model of **si**de chain conformations estimated by maximum **li**kelihood*. BASILISK represents all relevant variables in continuous space and thus avoids the problems that are due to the discretization used in rotamer libraries. Furthermore, BASILISK incorporates the *ø *(phi) and *ψ *(psi) angles. This makes it possible to condition the sampling upon the residue's backbone conformation, which is known to exert a strong influence on the side chain's conformation [3]. BASILISK represents all amino acids in a single probabilistic model, which is an entirely novel way of attacking this problem. Such an approach corresponds to a powerful machine learning technique called *multitask *or *transfer learning*, which often leads to better models with fewer parameters [23,24].

We first describe the BASILISK model, then proceed to evaluate its performance, and conclude with illustrating some potential applications.

## Results and discussion

### Parameterization

**Figure 1:**
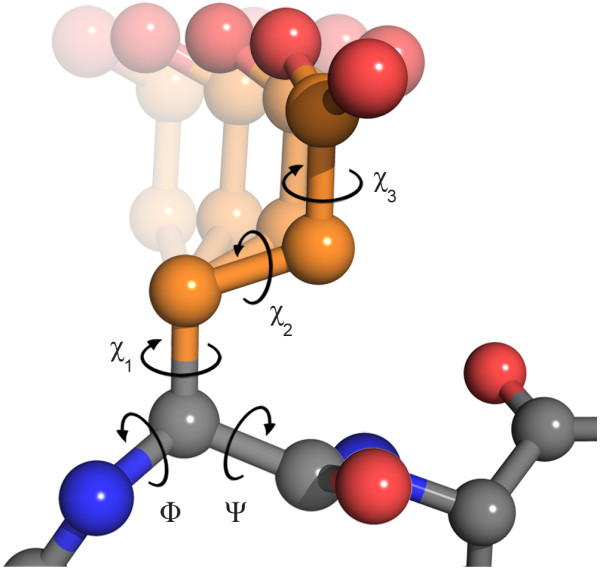
**Dihedral angles in glutamate**: Dihedral angles are the main degrees of freedom for the backbone (*ϕ *and *ψ *angles) and the side chain (*χ *angles) of an amino acid. The number of *χ *angles varies between zero and four for the 20 standard amino acids. The figure shows a ball-and-stick representation of glutamate, which has three *χ *angles. The fading conformations in the background illustrate a rotation around *χ*_1_. The figure was made using PyMOL http://www.pymol.org.

For our purposes, bond angles and bond lengths in amino acid side chains can be considered as fixed to their ideal values, as they show only very small variations [25]. This leaves the rotations around the bonds - the *χ *dihedral angles - as the main degrees of freedom. Accordingly, the sequence of *χ *angles is a good parameterization of the conformation of a given side chain. The same parameterization of the conformational space is also used in most popular rotamer libraries [6,7]. The number of angles necessary to describe a side chain conformation varies between zero and four for the 20 different standard amino acid types. Figure 1 illustrates the dihedral angles for glutamate.

In previous studies, it has been shown that the side chain conformation correlates highly with the conformation of the backbone [6]. For the backbone, the two main degrees of freedom are the *ϕ *and *ψ *dihedral angles, which when plotted against each other result in the celebrated Ramachandran plot [26]. We incorporate *ϕ *and *ψ *angles in BASILISK to be able to condition the sampling on the backbone conformation.

### BASILISK: a generative model of side chain conformations

Bayesian networks are graphical models that determine the possible factorizations of the joint probability distribution of a set of random variables [27-29]. A Bayesian network is a graph in which the nodes represent the random variables, and the edges encode their conditional independencies. The graph is directed and acyclic, and is often chosen based on prior, expert knowledge. If sequences of variables are modeled, the models are called *dynamic *Bayesian networks (DBNs) [22]. Here, *dynamic *originally referred to time sequences, such as speech signals, but arbitrary sequences can be modeled. Each position in the sequence is called a *slice*. The sequences represented by a DBN are allowed to have different lengths; a property which we use to our advantage, as explained below.

**Figure 2:**
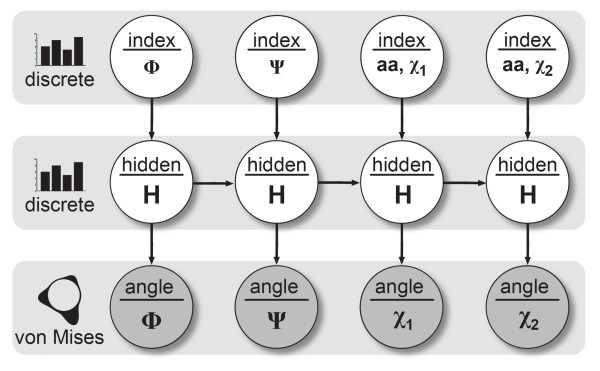
**The BASILISK dynamic Bayesian network**: The network shown represents an amino acid with two *χ *angles, such as for example histidine. In this case, the DBN consists of four slices: two slices for the *ϕ*, *ψ *angles, followed by two slices for the *χ *angles. Sampling a set of *χ *angles is done as follows. First, the values of the input nodes (top row) are filled with bookkeeping indices that determine both the amino acid type (for example histidine) and the labels of the angles (for histidine, *ϕ*, *ψ *followed by *χ*_1 _and *χ*_2_). In the next step, the hidden node values (middle row, discrete nodes) are sampled conditioned upon the observed nodes. These observed nodes always include the index nodes (top row, discrete nodes), and optionally also the *ϕ*, *ψ *nodes (first two nodes in the bottom row) if the sampling is conditioned on the backbone. Finally, a set of *χ *angles is drawn from the von Mises nodes (bottom row), whose parameters are specified by the sampled values of the hidden nodes.

BASILISK was implemented as a DBN, and its parameters were estimated by a maximum likelihood method using a data set derived from more than 1500 crystal structures (see Data sets for training and testing). The model is shown in Figure 2.

We briefly describe the most important features of the model. For each slice, an input node (which is a discrete variable) indicates which angle and amino acid type are modeled. For example, 3 indicates the *χ*_1 _angle of aspartate, 4 indicates the *χ*_2 _angle of aspartate, and so forth. We recently used a similar approach to formulate a probabilistic model of RNA structure [30].

The natural manifold of all the dihedral angles combined is the hypertorus: a torus with dimension three or higher. BASILISK constructs a probability distribution on this manifold in the following way: the model has a single output node per slice, in the form of a von Mises distribution [31], which can be considered the circular equivalent of a Gaussian distribution. The von Mises distribution takes the circular nature of the data into account: a dihedral angle in [- *π*, *π*[ is naturally represented as a point on the circle. The von Mises distribution belongs to the realm of directional statistics [31]: the statistics of angles, directions and orientations. We previously developed probabilistic models of RNA and protein structure based on the combination of DBNs and directional statistics [30,32,33]. The von Mises distribution was also used previously in a seminal study on probabilistic models of the protein backbone in terms of the *ϕ *and *ψ *angles [34], and in a preliminary study on clustering of side chainrotamers [35].

The problem of representing amino acid types that differ in the number of *χ *angles within one model is elegantly solved by using a DBN with a variable number of slices. There are two slices for the *ϕ *and *ψ *angles, followed by a sequence of slices that represent the *χ *angles. For example, to model glutamine, which has three *χ *angles in its side chain, one slice is added to the DBN that is shown in Figure 2.

The dependencies between the input nodes (which specify the amino acid type and the angles) and the output nodes (which specify the values of the angles) are mediated by a sequence of interconnected, discrete hidden nodes. The values of these nodes are never observed: their technical purpose is to model the dependencies between the amino acid type and the *χ *angles on the one hand, and the sequential dependencies between the *χ *angles on the other hand. The hidden nodes are so-called *nuisance variables*, that are integrated away in parameter estimation, sampling and inference. It should be noted that the hidden nodes thus introduce dependencies between *all *angles, and not just between two consecutive angles - a common misconception.

Our aim was to describe all side chain and backbone angles for all different amino acid types in a single probabilistic model. This approach is known as multitask or transfer learning in the field of machine learning [23,24] and has several advantages. As the same set of distributions is used to model all amino acids (and all angles), it leads to a lower amount of free parameters. Moreover, it makes "knowledge transfer" possible between amino acids with similar conformational properties during training [23,24]. Finally, for rotamer libraries, one needs to determine the optimal number of rotamers for each amino acid type separately, while in our approach, only the size of the hidden node needs to be determined. This can be done using an established statistical procedure for model selection (see Model training).

Several other network architectures were discarded during model selection as they could not adequately capture the joint distributions, which illustrates the importance of the network's architecture. Neither regular hidden Markov models, nor various mixture models produced satisfying results (data not shown). A similar observation was made for the probabilistic model of RNA structure mentioned previously [30].

It is important to note that the model as depicted in Figure 2 represents a single amino acid. When assigning side chains to an entire protein backbone, the model is simply applied to each position in the chain. Sampling conformations from BASILISK is fast: generating 50,000 arginine side chain conformations takes about two seconds on an average desktop computer (Intel Core 2, at 2.4 GHz).

### Probability distributions

The joint probability distribution encoded by the Bayesian network is

where  is the sequence of *χ *angles,  is the angle index information which includes the amino acid type, *ϕ *and *ψ *are the backbone angles, and  is the sequence of hidden node values.

For most purposes, the conditional and marginal distributions are of interest. BASILISK allows backbone dependent sampling of the *χ *angles by conditioning on the *ϕ *and *ψ *angles. The conditional probability distribution for this case is given by:

where the sums run over all possible hidden node sequences .

The marginal, conditional probability distribution for the backbone independent case is given by:

where the sum again runs over all possible hidden node sequences . The backbone angles are thus simply disregarded in this calculation.

The marginal, conditional probabilities *P*(*χ*|*H*) for a single angle are represented by von Mises distributions (see material and methods). The parameters of the von Mises distribution are specified by the value of the hidden node, *H*.

As BASILISK is a simple extension of a hidden Markov model, all relevant joint and marginal probabilities can be calculated in a straightforward manner using the forward algorithm [30,36].

### Initial analysis

For generative probabilistic models, an obvious first quality check is visual inspection. Accordingly, we start by investigating whether BASILISK captures the angular preferences found in the training data, before performing a more rigorous and in-depth analysis.

**Figure 3:**
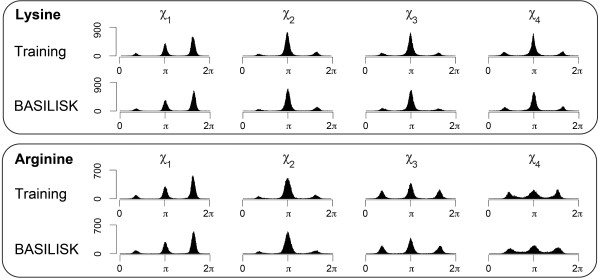
**Univariate histograms for lysine and arginine**: The histograms marked "Training" represent the training set. The histograms marked "BASILISK" represent BASILISK samples. For each amino acid, all histograms are plotted on the same scale.

For these first tests, we generated over 300,000 samples with the same amino acid composition as the training set. Figure 3 compares the marginal angular distributions of the training set with those of the BASILISK samples for arginine and lysine. We show plots for arginine and lysine because they are the only amino acids with four *χ *angles; they were most difficult to capture accurately with alternative models (data not shown). A comparison of all remaining relevant amino acids is available as additional material (Additional files 1, 2, 3, Figures S1-S3).

As a second test, we inspected pairwise histograms of the *χ*_1 _and *χ*_2 _angles for all relevant amino acids. These plots provide an indication of whether the model captures the correlation between two angles correctly. Again we compare samples from BASILISK model with the training data.

**Figure 4:**
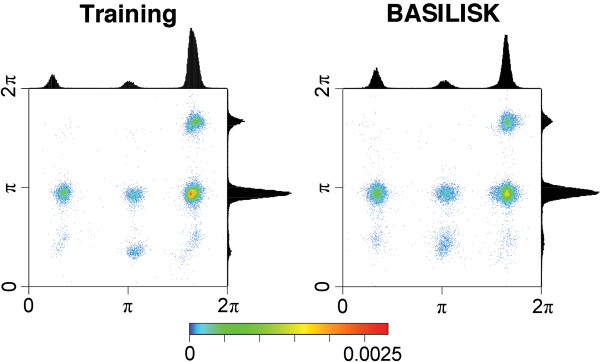
***χ*_1 _versus *χ*_2 _histogram for isoleucine**: The two-dimensional histogram of *χ*_1 _(x-axis) against *χ*_2 _(y-axis) illustrates the association between the two angles. Their univariate, marginal distributions are shown as well, attached to the respective axes. The histogram marked "Training" represents the training set, while the histogram marked "BASILISK" represents BASILISK samples.

Figure 4 shows the pairwise histograms for isoleucine. In the univariate, marginal plots for isoleucine, we observe three peaks for each *χ *angle. Hence, one might expect nine peaks with varying density in the bivariate plot. However, the training data only shows five major peaks. The comparison of the plots indicate that these features are indeed captured. Plots for the remaining 13 amino acid that have at least two *χ *angles are available as additional material (Additional files 4, 5, 6, Figures S4-S6).

The results of the visual inspections provide a strong indication that the model behaves as desired. We now move on to a more qualitative, in-depth analysis.

### BASILISK concurs with a standard rotamer library

In the following tests, we compare BASILISK to several rotamer libraries, as these are the method of choice to explore the conformational space of amino acid side chains for many purposes.

We use the Dunbrack backbone independent library [6] (bbind02.May.lib) as a representative rotamer library, as it is widely used and based on rigorous statistical analysis of high quality protein structures. In the construction of the Dunbrack library, it was assumed that the distribution of each angle in a rotamer follows a Gaussian distribution. Accordingly, rotamers in this library are reported as a sequence of Gaussian distributions - one for each *χ *angle. This allows us to evaluate the probability density value for any given side chain conformation according to the Dunbrack library.

For all calculations we used the BASILISK model with the backbone angles marked as unobserved - which results in a backbone independent likelihood - for fair comparison with the backbone independent Dunbrack rotamer library.

**Figure 5:**
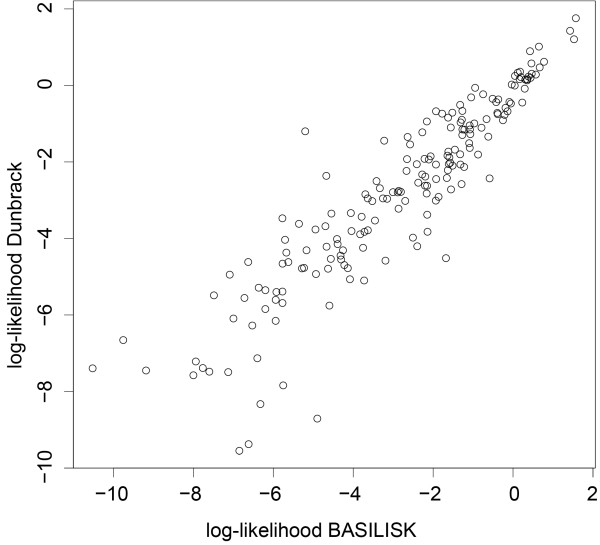
**Comparison between BASILISK and a standard rotamer library**: We calculated the log-likelihood for every rotamer in the Dunbrack backbone independent rotamer library according to the Gaussian model of the library itself (y-axis), and according to BASILISK (x-axis). The Pearson correlation coefficient is 0.91.

As a first test, we determine whether the Dunbrack rotamer library and BASILISK report similar probabilities for the same conformations. We calculated the log-likelihood of the side chain conformations according to the Dunbrack backbone independent rotamer library and according to BASILISK for all side chains in the test set (see Data sets for training and testing). The results show that the two methods indeed correlate very well (Pearson correlation coefficient is 0.88). Figure 5 shows a scatter-plot of the log-likelihood values for all rotamer conformations in the Dunbrack library according to the library itself, and according to BASILISK. Again we find a very good correlation (Pearson correlation coefficient is 0.91). Outliers, especially in the low probability region, are limited to very rare rotamer conformations with little to no observations according to the Dunbrack library.

In the following paragraphs we take a more detailed look at the probability distributions encoded in the BASILISK model, and compare with several standard rotamer libraries. For this analysis we again use the Dunbrack library, but also include two additional backbone independent libraries [7,37].

The Kullback-Leibler (KL) divergence is a standard measure of the similarity between two probability distributions [28,38]. Here, the KL divergence is used to compare the quality of the rotamer libraries versus BASILISK. We compare BASILISK and the rotamer libraries with the experimental data in the test, which is here used as a reference or truth model. We then calculate the relative KL divergence between BASILISK and each library (see Kullback-Leibler divergence). Table 1 shows the results for each amino acid. Positive numbers indicate that BASILISK models the conformations in the test set more accurately than the method compared to, while negative numbers indicate that BASILISK is less accurate.

**Table 1:** Kullback-Leibler divergence analysis.

Amino acid	Dunbrack	Tuffery	Lovell	BASILISK backbone-dependent
Cysteine	0.01	0.03	0.00	-0.13
Aspartate	0.27	0.53	0.79	-0.49
Glutamate	0.16	0.89	1.16	-0.16
Phenylalanine	0.04	0.49	0.20	-0.35
Histidine	0.88	1.98	1.56	-0.10
Isoleucine	0.13	0.02	1.15	-0.43
Lysine	0.38	0.72	6.55	-0.23
Leucine	0.10	0.32	2.38	-0.21
Methionine	0.00	0.22	2.91	-0.06
Asparagine	0.42	0.82	3.60	-0.41
Glutamine	0.28	1.00	6.41	-0.17
Arginine	0.15	0.80	2.59	-0.17
Serine	0.04	0.16	0.10	-0.22
Threonine	0.06	0.14	0.25	-0.48
Valine	-0.07	0.13	-0.06	-0.45
Tryptophan	-0.33	0.07	1.49	-0.04
Tyrosine	-0.06	0.37	0.21	-0.33

Average	0.14	0.46	1.69	-0.28

Columns 2-4: the differences in the KL divergences between BASILISK without backbone information and a set of standard rotamer libraries. Last column: the differences in the KL divergences between backbone independent BASILISK and BASILISK including backbone information. In both cases, positive numbers indicate that backbone independent BASILISK captures the observed preferences in the test set more accurately.

Overall, BASILISK captures the conformational preferences of amino acid side chains more accurately than any of the rotamer libraries, with few exceptions. For example, the Dunbrack library performs better for tryptophan. We speculate this is due to the relatively low abundance of tryptophan in the training data (< 2%), and the fact that we are training an all amino acid model. An amino acid with few observed data points will have a smaller impact on the estimated parameters than an amino acid that was presented more often during training. The relatively low performance of the Lovell library can be explained by the small number of rotamers in the library. Especially for long side chains such as glutamine, lysine or arginine, too many rotamers appear to be missing.

Note that only libraries reporting both mean and variance for each *χ *angle are suitable for this analysis. Many rotamer libraries are mere discrete sets of possible conformations [8,9]: they cannot be evaluated in the same, rigorous way.

### BASILISK captures the backbone's influence

It is well known that there is a strong correlation between backbone and side chain conformations [3]. This backbone dependency is captured by the BASILISK model by incorporating the backbone's two main degrees of freedom: the *ϕ *and *ψ *dihedral angles.

To the best of our knowledge, BASILISK is the first model that captures this correlation in continuous space; that is, without resorting to the usual discretizations of the conformational space. Comparison with other models is therefore difficult. Notably, a comparison with backbone dependent rotamer libraries based on the KL divergence is not possible due to their lack of an expression for the joint probability of the backbone and the side chain angles. However, we do present two decisive tests. First, we resort to visual inspection, followed by a quantitative evaluation based on the KL-divergence between backbone dependent and independent BASILISK. In the next section, we evaluate the influence of the backbone information on side chain sampling for a fixed backbone.

**Figure 6:**
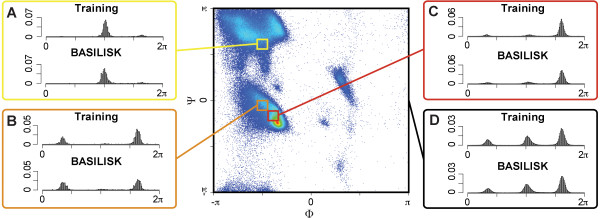
**Backbone dependency of the *χ*_1 _angle of aspartate**: The *χ*_1 _histograms for different areas of the Ramachandran plot indicate a strong correlation between the backbone and the side chain conformations. For some regions certain peaks disappear almost entirely. Histograms marked with "Training" represent the training set, and histograms marked "BASILISK" represent BASILISK samples. A, B and C show the histograms for the areas indicated by the three boxes, while D shows the histogram over the entire space. Note that the indicated binning of the backbone space is for visualization only, as BASILISK does not rely on discretization of the conformational space.

Figure 6 shows a comparison between the angular preferences observed in the training data and those found in BASILISK samples. For this comparison, we generated BASILISK samples and binned them according to their backbone angle values. The bins chosen in the plot are well populated in the training set, and illustrate the drastic effect that the backbone conformation can have on the side chain's conformational space. Certain peaks are almost entirely missing for certain regions of the Ramachandran plot. These observations provide a first indication that BASILISK captures the correlation with the backbone well.

For a quantitative evaluation, we turn again to the KL-divergence; this time, to compare BASILISK with and without backbone dependency. As in the previous test, where BASILISK was compared to different rotamer libraries, this test determines which model is closer to the theoretical truth model as embodied by the test set (see Data sets for training and testing). Table 1 (last column) shows the differences between the KL divergences for all relevant amino acids. The negative numbers indicate that the backbone dependent model resembles the experimental data most. This indicates that BASILISK indeed captures the influence of the backbone, and that incorporating backbone information improves the accuracy for all amino acids. This conclusion is also supported by the results described in the next section.

### Application: side chain sampling for a fixed backbone

In the previous sections, we evaluated the probability distributions encoded in BASILISK. We now proceed to evaluate the applicability of the model. As a proof of concept we implemented a side chain prediction algorithm that assigns side chains to a fixed protein backbone. Xiang and Honig [8] showed that given a large rotamer library, an MCMC approach leads to good accuracy in predicting buried side chain conformations. Following their study, we combine side chain sampling from BASILISK with an MCMC method and an unmodified Lennard-Jones potential [39]. The main purpose of the potential is to avoid steric clashes between the side chains. The parameters for the potential were derived from the OPLS force field as implemented in Tinker [40,41].

For the evaluation, we considered a *χ *angle within ± 20° of its value in the crystal structure as correct [8,12]. We only included buried side chains in the protein core in the evaluation, because exposed side chains often have few steric restraints and we did not use any energy term that accounts for the solvent interaction. However, all side chains were included in the MCMC procedure and none were fixed, in order not to bias the simulation towards the native state. The test set contained 43 single chain crystal structures that were used to evaluate side chain prediction algorithms in other studies [8,9,42].

To avoid getting trapped in local minima, we resampled three random residues at a time [11]. This effectively enables side chains to swap their positions. After a fixed set of MCMC steps, the lowest energy structure was selected as the final prediction. For details on the energies and the sampling see Sampling strategy and energies.

BASILISK is a true probabilistic model: it can be used as a proposal distribution to implement MCMC methods that respect detailed balance, since its exact contribution can be taken into account. The first test uses a Lennard-Jones potential as the only energy component, and brings in BASILISK solely as a proposal function. We use the BASILISK model to propose new side chain conformations, which are subsequently accepted or rejected based on their energy (see Sampling strategy and energies). Table 2b shows that already with this very simple approach, we reach a quite reasonable performance: more than 87% of the *χ*_1 _angles were correctly predicted (Table 2b).

**Table 2:** Side chain placement for a fixed backbone.

Structure	L, B^*a*^	Lennard-Jones^*b*^		BASILISK^*c*^		BASILISK BB^*d*^		IRECS^*e*^		SCWRL 4^*e*^	
		*χ*_1_(%)	*χ*_2_|*χ*_1 _(%)^*f*^	*χ*_1 _(%)	*χ*_2_|*χ*_1 _(%)^*f*^	*χ*_1 _(%)	*χ*_2_|*χ*_1 _(%)^*f*^	*χ*_1 _(%)	*χ*_2_|*χ*_1 _(%)^*f*^	*χ*_1 _(%)	*χ*_2_|*χ*_1 _(%)^*f*^
1NAR	289, 45	77.78	76.92	73.33	87.50	93.33	87.50	84.44	70.37	86.67	79.31
5P21	166, 21	90.48	77.78	85.71	100.00	95.24	88.89	95.24	88.89	100.00	80.00
1BJ7	150, 12	91.67	75.00	100.00	77.78	100.00	77.78	91.67	75.00	83.33	85.71
1GCI	269, 40	95.00	86.67	97.50	81.25	100.00	81.25	95.00	85.71	97.50	93.33
2BAA	243, 30	86.67	60.00	83.33	86.67	90.00	82.35	86.67	81.25	90.00	94.12
1DHN	121, 15	93.33	100.00	100.00	85.71	86.67	100.00	93.33	100.00	93.33	100.00
1AMM	174, 22	90.91	100.00	100.00	92.86	100.00	92.86	100.00	85.71	86.36	90.91
1IC6	279, 47	89.36	73.68	91.49	70.00	93.62	75.00	91.49	73.68	93.62	85.00
1KOE	172, 26	80.77	92.86	73.08	84.62	80.77	84.62	92.31	92.86	88.46	84.62
2HVM	273, 43	83.72	74.07	95.35	82.76	90.70	89.66	90.70	85.19	90.70	84.62
3LZT	129, 10	80.00	83.33	70.00	83.33	80.00	83.33	100.00	83.33	100.00	83.33
1CEX	197, 24	95.83	89.47	100.00	100.00	95.83	100.00	87.50	94.74	100.00	100.00
1AGY	197, 25	92.00	84.21	96.00	100.00	96.00	100.00	84.00	94.74	92.00	100.00
1EDG	380, 76	85.53	65.85	85.53	85.00	90.79	81.82	85.53	80.49	89.47	86.05
1THV	207, 20	85.00	76.92	80.00	91.67	85.00	92.31	90.00	76.92	90.00	100.00
2CPL	164, 24	95.83	73.33	91.67	85.71	95.83	93.33	100.00	80.00	95.83	93.33
1NPK	150, 16	100.00	60.00	100.00	80.00	100.00	90.00	93.75	88.89	87.50	88.89
1MLA	305, 38	97.37	71.43	97.37	86.36	100.00	86.36	94.74	75.00	92.11	84.21
1ARB	263, 37	89.19	90.48	89.19	88.89	86.49	85.00	86.49	68.42	83.78	90.00
1MML	251, 19	78.95	81.82	84.21	75.00	89.47	91.67	94.74	83.33	94.74	91.67
1CHD	198, 26	96.15	68.75	96.15	81.25	100.00	76.47	92.31	73.33	92.31	80.00
1QQ4	198, 25	80.00	60.00	84.00	80.00	84.00	90.91	88.00	63.64	88.00	81.82
1QJ4	256, 36	83.33	75.00	91.67	81.48	94.44	81.48	91.67	85.71	94.44	81.48
1B9O	123, 14	78.57	50.00	71.43	57.14	78.57	50.00	92.86	66.67	92.86	44.44
1BYI	224, 30	80.00	86.67	100.00	100.00	96.67	94.12	90.00	87.50	93.33	100.00
1XNB	185, 22	90.91	91.67	100.00	91.67	95.45	100.00	100.00	66.67	95.45	91.67
2PTH	193, 19	94.74	100.00	100.00	100.00	94.74	100.00	94.74	100.00	100.00	100.00
1WHI	122, 12	91.67	100.00	100.00	75.00	100.00	75.00	91.67	100.00	100.00	100.00
153L	185, 26	84.62	93.33	96.15	93.33	88.46	86.67	100.00	87.50	100.00	100.00
1QTW	285, 35	94.29	84.00	91.43	91.67	91.43	83.33	88.57	70.83	97.14	80.77
2IHL	129, 12	66.67	75.00	75.00	75.00	83.33	75.00	100.00	75.00	100.00	75.00
1CZ9	139, 14	85.71	88.89	85.71	100.00	100.00	100.00	100.00	88.89	100.00	100.00
2RN2	155, 11	90.91	87.50	90.91	87.50	90.91	100.00	81.82	100.00	81.82	100.00
1RCF	169, 28	92.86	87.50	96.43	82.61	100.00	95.83	82.14	80.00	92.86	90.91
7RSA	124, 12	75.00	100.00	75.00	100.00	75.00	100.00	75.00	100.00	91.67	100.00
1A8Q	274, 39	87.18	70.00	71.79	93.75	89.74	80.95	94.87	87.50	92.31	95.65
1CEM	363, 55	89.09	64.52	83.64	77.42	94.55	79.41	89.09	80.00	89.09	80.00
1AAC	105, 13	100.00	75.00	100.00	100.00	100.00	100.00	92.31	87.50	100.00	87.50
1PLC	99, 10	100.00	85.71	90.00	66.67	100.00	71.43	90.00	85.71	100.00	71.43
1BD8	156, 11	81.82	100.00	90.91	85.71	90.91	100.00	100.00	100.00	90.91	85.71
1NLS	237, 23	78.26	85.71	86.96	87.50	86.96	81.25	86.96	80.00	91.30	81.25
1IXH	321, 44	81.82	72.00	84.09	84.62	88.64	85.19	93.18	75.00	88.64	84.00
1AKO	268, 35	77.14	72.73	85.71	91.30	85.71	91.30	100.00	81.48	97.14	88.46

Average	207, 27	87.44	80.65	89.32	86.25	92.08	87.49	91.92	83.43	93.13	88.26

The table shows the percentage of correctly predicted *χ *angles for the different MCMC experiments. ^*a *^number of residues (L), number of buried residues (B), ^*b *^using the Lennard-Jones energy and BASILISK as a proposal distribution, ^*c *^using BASILISK without backbone information, ^*d *^using BASILISK with backbone information (BB), ^*e *^using IRECS or SCWRL with default parameters, ^*f *^percentage of correctly predicted *χ*_2 _angles given a correctly predicted *χ*_1 _angle.

In a second test, we use the BASILISK likelihood as a pseudo-energy component [43], combined with the Lennard-Jones potential. Table 2c shows that the prediction quality increases (by about 2% for *χ*_1_). Incorporating backbone information in BASILISK (Table 2d) increases the performance compared to the backbone independent (by more than 2%) and the Lennard-Jones only case (by more than 4%). It should be noted that none of these tests include energies that evaluate key features such as hydrogen bonds, salt-bridges or electrostatic interactions.

For comparison, Table 2e also reports the prediction accuracy of IRECS [12] and SCWRL 4 [14], which are two leading programs in the field. In both cases, the results are on a par with BASILISK. SCWRL 4 and IRECS are specialized programs optimized for both prediction accuracy and speed: they use fine tuned and optimized force fields combined with backbone dependent rotamer libraries. The comparison serves to illustrate the quality of BASILISK as a probabilistic model; with respect to computational performance, SCWRL 4 and IRECS (whose runtime is typically seconds to minutes) are clearly superior to the unoptimized application of BASILISK (where we sampled for several hours to ensure convergence).

## Conclusions

In this paper, we introduce a generative, probabilistic model of the conformational space of side chains that allows sampling of realistic, native-like side chain angles in continuous space. BASILISK incorporates a continuous backbone dependency, which to the best of our knowledge is entirely novel. Another unique feature is that BASILISK represents all relevant natural amino acids within one model. This powerful approach is known as multitask or transfer learning in the field of machine learning, and comes with several advantages.

In the first tests we showed that BASILISK is able to accurately capture the angular preferences found in proteins. Using the KL divergence, we confirmed that the model compares favorably with several standard rotamer libraries.

BASILISK also captures the effect of the backbone conformation on the side chain, and samples realistic angular distributions for various areas of the Ramachandran plot. Again using the KL divergence, we confirmed that including backbone information leads to more accurate results.

As a proof of concept, we implemented a simple side chain prediction method that assigns side chains to a fixed protein backbone, using an MCMC sampling scheme and an unmodified Lennard-Jones potential as energy function. The results show the applicability of combining a continuous description of conformational space with a detailed energy function. Adding BASILISK as an explicit pseudo-energy term improves the prediction results. Best results are obtained when the backbone conformation is taken into account.

Specialized side chain prediction programs combine highly tuned energy functions with an efficient exploitation of the discrete nature of rotamers. This makes them very fast, yet accurate. BASILISK as such does not compete with those programs, but provides a solution where a detailed, continuous description of conformational space is required. The possibility to combine an unmodified, standard force field with BASILISK opens great possibilities for applications such as docking, structure prediction or protein design. For example, the calculation of side chain entropies is important for tasks such as protein quality assessment or protein design [44-46]. BASILISK can facilitate these calculations - which are now often performed with discrete rotamer libraries - in continuous space.

BASILISK, when combined with our previously described probabilistic model of the protein backbone (TorusDBN [33]), can be used to sample protein structures in continuous space and in full atomic detail. An obvious potential application is to sample protein conformations by combining TorusDBN and BASILISK with a physical force field, with applications in protein structure prediction, simulation and design.

BASILISK illustrates the enormous potential and increasing importance of probabilistic models and probabilistic machine learning methods in structural bioinformatics [19,30,33,44,47,48]. We believe that BASILISK is an excellent solution for problems that require going beyond discrete representations of amino acid side chains in protein structure simulation, prediction and design.

## Methods

### Data sets for training and testing

For training, we used a high quality dataset obtained from the PISCES server [49]. The PISCES server can be used to select a set of structures from the Protein Data Bank that respect quality thresholds regarding resolution or R-value. We imposed a resolution of 1.6 Å or better, an R-value of 0.25 or better, and a pairwise sequence similarity cutoff of 25%. Subsequently, all structures were removed that had a sequence similarity higher than 25% with any of the structures used in the fixed backbone prediction test (see next paragraph).

The resulting set consisted of 1703 crystal structures. All structures were processed using the REDUCE program [50], which automatically corrects some common errors in protein structures (such as histidines with a flipped ring). From this initial set retrieved from the PISCES server, we randomly picked 10% of the structures and reserved these for testing. The test set contained 31,229 side chains from 171 structures. The training set contained angles from 277,975 residues from 1532 structures. The angles were calculated using Biopython's Bio.PDB module [51,52].

### Data set for fixed backbone prediction

The data set for the fixed backbone prediction consisted of crystal structures that were previously used in three other studies for the same purpose [8,9,42].

We removed structures with a very small core (less than 10 fully buried residues) and structures with multiple chains or chain breaks, resulting in 43 remaining structures. The input files for the test only retained the backbone atoms; all side chain atoms and hetero-atoms were removed.

We used DSSP [53] to calculate the relative accessible surface area in order to determine the solvent exposure state of a residue. We only included fully buried residues for which the accessible surface area is zero in our analysis, as we did not use any energy term accounting for the solvent interaction. However, all side chains were included in the experiments, in order to avoid bias towards the native structure.

### Model training

The BASILISK model was implemented and trained using our freely available Mocapy ++ toolkit [54]. The model's parameters were estimated using the stochastic expectation maximization (EM) algorithm [55].

**Figure 7:**
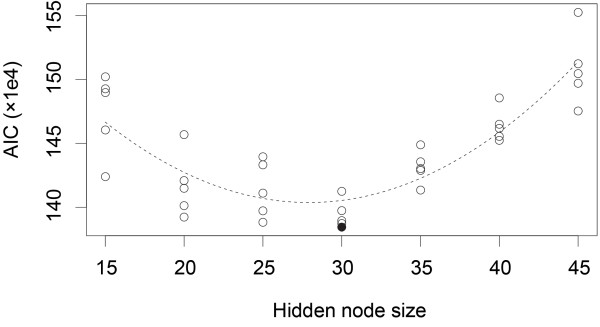
**Selecting the optimal model**: The Akaike information criterion (AIC) is used to determine the optimal number of hidden node values. The AIC score (y axis) points towards a model with 30 hidden node values (x-axis). The optimal model is shown as a filled circle.

The number of hidden node values (that is, the *size *of the hidden node) is a hyperparameter that has to be determined separately. For that purpose, we trained 5 models for each hidden node size (15, 20, 25, 30, 35, 40 and 45) and choose the model with the lowest Akaike information criterion (AIC) score [56]. The AIC score is defined as

where *L*(Θ|) is the likelihood of the trained model Θ given the observations , and *k *is the number of free parameters. The AIC is a measure of the estimated KL divergence between the trained model and a fictitious truth model which generated the training data [56]. In our case, the AIC points towards a model with 30 hidden node states (see Figure 7). The number of relevant, none-zero parameters for the selected model is 9871.

The model also includes backbone information, which usually leads to a quick explosion of parameters due to discretization. For example, dividing the Ramachandran plot in bins of 10° by 10° creates 1296 bins, which combined with 18 rotameric amino acids and several rotamers per amino acid can quickly lead to data sparseness in training. This problem is avoided by treating the data in a natural, continuous space.

### Modeling the angles

We use the von Mises distribution [31] to model both the backbone and the side chain angles. This distribution can be considered as the circular equivalent of the univariate Gaussian distribution. As an angle can be represented as a point on the circle, it is the correct approach to use a probability distribution that takes the circularity of the data into account. The probability density function of the von Mises distribution has the following form:

where *x *∈ [- *π*, *π*[ is the random angle, *μ *is the mean, *κ *≥ 0 is a concentration parameter and *I*_0 _is the modified Bessel function of the first kind and of order zero.

### Probability densities from rotamer libraries

For the calculation of the KL divergences, we need the probability density of a conformation according to the different rotamer libraries. According to a typical rotamer library, the probability density of a given angle sequence is:

where  is the sequence of *χ *angles, *A *is the amino acid type, *P*(*R*_*A*_) is the probability of the rotamer *R *of amino acid type *A*, and *P*(|*R*_*A*_) is the probability of the sequence of *χ *angles for rotamer *R *of amino acid type *A*. The sum runs over all defined rotamers for the amino acid A in a rotamer library.

Each individual angle *χ*_*n *_in a rotamer *R*_*A *_is distributed according to a Gaussian distribution , with mean  and standard deviation . Hence, in the above expression, the conditional probability density of the sequence of angles is equal to the product of the probability densities of the individual angles:

The product runs over all *n χ *angles.

### Kullback-Leibler divergence

In the evaluation of the model, we used the KL divergence [38] to compare BASILISK with various rotamer libraries. For two probability density functions, the KL divergence is defined as

where *p *is usually the empirical data or a truth model and *q *usually represents a probabilistic model that approximates *p*. The KL divergence is non-negative and only becomes zero if *p *= *q*.

In order to evaluate whether BASILISK captures the angular preferences more accurately than a standard rotamer library, we calculate the difference between the KL divergence from the experimental data to a rotamer library, and the KL divergence from the experimental data to BASILISK:

where *q*_R _is the probability distribution associated with the rotamer library, *q*_B _is the BASILISK model and *p *is given by the experimental data. In order to calculate this difference, we use the fact that the relative KL divergence can be expressed as a statistical expectation [30,56] leading to:

where  is the expectation with respect to *p*. In our case, the empirical distribution *p *is a set of *n *observations *x*_1_, *x*_2_, ... *x*_*n*_. Hence, we can calculate the expectation by averaging over these observations:

For the backbone dependent case, a fair evaluation based on the KL divergence is not possible: rotamer libraries do not allow the calculation of the joint probability of the *x *, *ϕ *and *ψ *angles.

### Sampling strategy and energies

For the fixed backbone test, we used a Lennard-Jones 6-12 potential. The energy for an atom pair is:

where *d *is the distance between two atoms, *ε *is the depth of the attractive well and *σ *is the optimal distance between two atoms based on their van der Waals radii. The atom dependent *ε *and *σ *values were taken from the OPLS/Tinker parameter set [40,41]. Subsequently, we used Boltzmann's law to turn the energies into probabilities:

where *E*_LJ_() is the Lennard-Jones energy of conformation , *R *is the ideal gas constant and *T *is the temperature. We set the temperature to room temperature, which results in  for all calculations.

For sampling, we use the classic Metropolis-Hastings MCMC approach [28]:

where  is the probability of accepting to replace  with ;  and  are the probabilities of  and  according to the target distribution; and  and  are the probabilities to move to state  from state  and to state  from state  according to the proposal distribution.

In the first test, we use the Lennard-Jones term as the only energy component, and use BASILISK solely as a proposal distribution:

where  is the Lennard-Jones derived probability of  as before, and  is the product of the probabilities of the side chains in  according to BASILISK. Note that in this case, we sample according to the Lennard-Jones potential in an unbiased way.

For the second test, we included BASILISK as an explicit pseudo-energy term. We approximate the probability of seeing a certain state  as the product of the Lennard-Jones derived probability, , with the probability of the side chain conformation according to BASILISK, :

Because BASILISK is used both as a proposal distribution and a pseudo-energy term, the Metropolis-Hasting expression reduces to:

For the backbone dependent experiments,  is replaced in the above expressions by , where  and  are the (fixed) backbone angles of .

We proposed three new side chain conformations at random sequence positions in every iteration. This effectively enables side chains to swap places, which is important to solve the combinatorial problem in the densely packed protein core. The backbone independent sampling is done using ancestral sampling [28]. In the backbone dependent case, sampling is done using the forward-backtrack algorithm [30,32,33,57].

In the fixed backbone experiments, we used 500,000 MCMC iterations. For the final prediction, we selected the structure with the highest probability (or, equivalently, lowest energy). In the evaluation, we considered a *χ *angle within 20° of the angle observed in the crystal structure as correct.

## Availability

A Python implementation of BASILISK, including all the parameters of the optimal model, is freely available for download at https://sourceforge.net/projects/basilisk-dbn/. The source code provides a class that implements the main features of BASILISK, namely sampling side chain angles and evaluating the probability of a given set of angles. The package also includes example scripts that show how to use the BASILISK module. The first script parses a Protein Data Bank file [58] using Biopy-thon's Bio.PDB package [51,52], retrieves all the side chains and calculates the likelihood of their *χ *angles. A second script samples side chain angles for a given amino acid type and optionally a set of backbone angles. For a full description of the features, please refer to the provided manual.

## Authors' contributions

TiH carried out the study. WB assisted in implementing the MCMC framework. MP and JF assisted in using the Mocapy++ toolkit. KEJ implemented the OPLS force field. TH conceived the study. TH and TiH wrote the manuscript. All authors read and approved the final manuscript.

## Supplementary Material

Additional file 1 **Univariate histograms for all amino acids with one *χ *angle**. Histograms marked "Training" were generated from the training set; histograms marked "BASILISK" were generated from BASILISK samples. Click here for file

Additional file 2 **Univariate histograms for all amino acids with two *χ *angles**. Histograms marked "Training" were generated from the training set; histograms marked "BASILISK" were generated from BASILISK samples. Click here for file

Additional file 3 **Univariate histograms for all amino acids with three *χ *angles**. Histograms marked "Training" were generated from the training set; histograms marked "BASILISK" were generated from BASILISK samples. Click here for file

Additional file 4 ***χ*_1 _versus *χ*_2 _histograms for histidine, phenylalanine, glutamate, aspartate and proline**. Histograms marked "Training" were generated from the training set; histograms marked "BASILISK" were generated from BASILISK samples. Click here for file

Additional file 5 ***χ*_1 _versus *χ*_2 _histograms for asparagine, leucine, methionine and lysine**. Histograms marked "Training" were generated from the training set; histograms marked "BASILISK" were generated from BASILISK samples. Click here for file

Additional file 6 ***χ*_1 _versus *χ*_2 _histograms for arginine, tyrosine, tryptophan and glutamine**. Histograms marked "Training" were generated from the training set; histograms marked "BASILISK" were generated from BASILISK samples. Click here for file
