# Supplementary material for: Beyond rotamers: a generative, probabilistic model of side chains in proteins
Source: BMC Bioinformatics. 2010 Jun 5;11:306. doi: 10.1186/1471-2105-11-306 (PMC2902450; doi:10.1186/1471-2105-11-306)
Supplement: Additional file 2 — Univariate histograms for all amino acids with two χ angles. Histograms marked "Training" were generated from the training set; histograms marked "BASILISK" were generated from BASILISK samples. [file 1471-2105-11-306-S2.PDF]

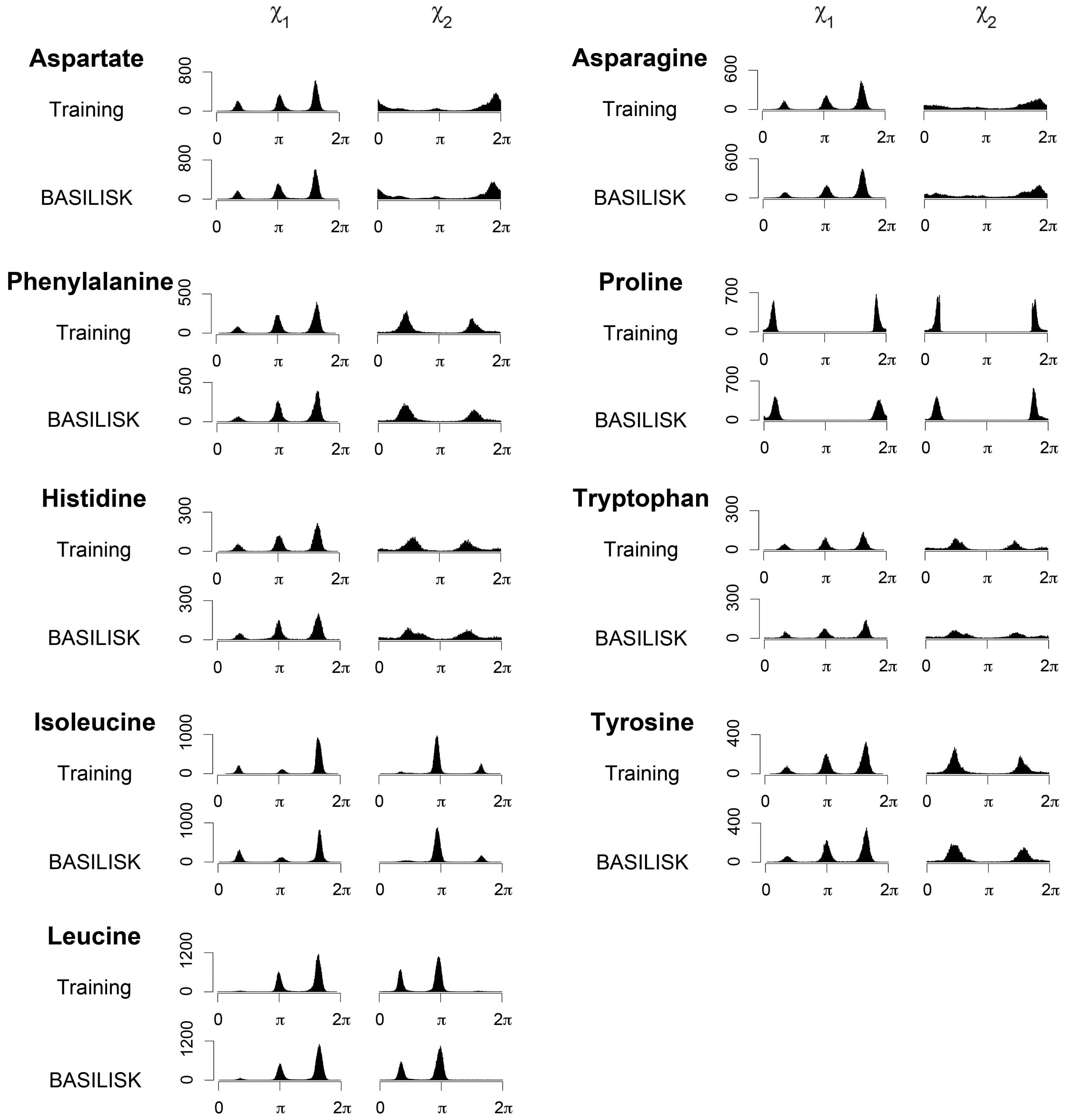

Supplementary figure 2: Univariate histograms for all amino acids with two χ angles. Histograms marked "Training" were generated from the training set; histograms marked "BASILISK" were generated from BASILISK samples.
